# Combining lipidomics and machine learning to measure clinical lipids in dried blood spots

**DOI:** 10.1007/s11306-020-01703-0

**Published:** 2020-07-24

**Authors:** Stuart G. Snowden, Aniko Korosi, Susanne R. de Rooij, Albert Koulman

**Affiliations:** 1 Core Metabolomics and Lipidomics Laboratory, Metabolic Research Laboratories, Institute of Metabolic Science, University of Cambridge, Level 4 Pathology, Cambridge Biomedical Campus, Cambridge, CB2 0QQ UK; 2 Centre for Neuroscience, Swammerdam Institute for Life Sciences, University of Amsterdam, Amsterdam, The Netherlands; 3 Department of Clinical Epidemiology, Amsterdam University Medical Centre, Biostatistics & Bio informaticslocation AMC, Amsterdam, The Netherlands; 4 Department of Public Health, Amsterdam University Medical Center, University of Amsterdam, Amsterdam, The Netherlands

**Keywords:** Lipidomics, Total cholesterol, Triglyceride, HDL, LDL

## Abstract

**Introduction:**

Blood-based sample collection is a challenge, and dried blood spots (DBS) represent an attractive alternative. However, for DBSs to be an alternative to venous blood it is important that these samples are able to deliver comparable associations with clinical outcomes. To explore this we looked to see if lipid profile data could be used to predict the concentration of triglyceride, HDL, LDL and total cholesterol in DBSs using markers identified in plasma.

**Objectives:**

To determine if DBSs can be used as an alternative to venous blood in both research and clinical settings, and to determine if machine learning could predict ‘clinical lipid’ concentration from lipid profile data.

**Methods:**

Lipid profiles were generated from plasma (n = 777) and DBS (n = 835) samples. Random forest was applied to identify and validate panels of lipid markers in plasma, which were translated into the DBS cohort to provide robust measures of the four ‘clinical lipids’.

**Results:**

In plasma samples panels of lipid markers were identified that could predict the concentration of the ‘clinical lipids’ with correlations between estimated and measured triglyceride, HDL, LDL and total cholesterol of 0.920, 0.743, 0.580 and 0.424 respectively. When translated into DBS samples, correlations of 0.836, 0.591, 0.561 and 0.569 were achieved for triglyceride, HDL, LDL and total cholesterol.

**Conclusion:**

DBSs represent an alternative to venous blood, however further work is required to improve the combined lipidomics and machine learning approach to develop it for use in health monitoring.

**Electronic supplementary material:**

The online version of this article (10.1007/s11306-020-01703-0) contains supplementary material, which is available to authorized users.

## Introduction

Traditionally, in high-income countries there is significant infrastructure to facilitate preclinical population health monitoring and research that is not present in low income countries leaving a significant unmet need for research and health monitoring (Kreuter et al. 2016; Johannessen 2010). Collecting samples in a hospital setting is still relatively straight forward, however owing to the difficulty, expense (relative to income) and risk of infection only individuals with serious or life-threatening symptoms are usually taken to hospital meaning that a sample cohort collected in a hospital alone would be biased.

In low income settings sending teams into the community to collect samples is one part of the solution to this. However, venous blood samples will need to be processed and frozen within a few hours of sample collection (Yin et al. 2015) and in rural areas of an LIC access to laboratory facilities to do this is unlikely to be available. It is with these limitations in mind that dried blood spots represent an attractive alternative to venous blood collection as it enables samples to be collected in remote geographic locations because processing the sample for storage does not require laboratory facilities (Spooner et al. 2019; Li and Tse 2010). The ability to collect smaller blood volumes is also attractive for working with fragile patient populations in LIC settings e.g. malnourished infants (Spooner et al. 2019; Lakshmy et al. 2014; Komada et al. 2015). In addition to this Prentice et al. (Prentice et al. 2013) showed that whilst room temperature storage of DBS samples for two years does lead to significant differences in metabolite composition, room temperature storage for just a week has very little effect, meaning that there can be a short delay between sample collection and storage at either − 20 °C or − 80 °C. These findings are consistent with other studies that have shown that a range of analytes including lipids, HIV antibodies and drugs including ceftriaxone and cocaine are stable on DBS cards stored at room temperature for short periods (Kyle et al. 2017; Page-Sharp et al. 2016; Yassin et al. 2013; Alfazil and Anderson 2008). In the context of research, where large sample cohorts can take several years to collect and especially when repeat sampling is involved, the effect of long term storage at − 80 °C is also an important experimental factor to consider. Prentice et al. also looked at the stability of lipid analytes in DBS stored at both − 20 °C and − 80 °C and demonstrate that storage at − 80 °C for 2 years had a minimal effect on the lipid composition of the DBS samples (Prentice et al. 2013).

For both research and health care monitoring it is important to determine if there is good concordance in the concentrations measured in both DBS and plasma samples. There is a significant body of literature looking at how to analyse a wide range of drug analytes in these samples (Wilhelm et al. 2014; Elst et al. 2013; Manicke et al. 2011), with DBS samples being standardly used pharmokinetic studies in neonates and young children (Abu-Rabie and Spooner 2009). An array of endogenous compounds have also been measured from dried blood spots including HbA1c (Crimmins et al. 2014; Lacher et al. 2013), total cholesterol (Abhijit et al. 2016; Miller et al. 2015; Corso et al. 2016), HDL (Lacher et al. 2013; Miller et al. 2015; Samuelsson et al. 2015), triglyceride (Lakshmy et al. 2012,2010; Quraishi et al. 2006) and CRP (Elst et al. 2013) among others. A detailed discussion of this literature is beyond the scope of this manuscript and there are several excellent reviews on this subject (Spooner et al. 2019; Zakaria et al. 2016; Chance et al. 2014).

Many of these studies have been successful and achieved good concordance between the concentration of a given analyte when measured in both DBS and plasma samples making them useful tools in research and healthcare monitoring. For example Lakshmy et al. was able to achieve a correlation of r = 0.797 between concentrations of total cholesterol measured in DBS and serum samples (Lakshmy et al. 2012). This is broadly in keeping with the other results reported in the literature with Samuelsson et al. reporting r = 0.480 (Samuelsson et al. 2015), Corso et al. achieving r = 0.611 (Corso et al. 2016) and Lacher et al. an R^2^ of 0.340 (Lacher et al. 2013) between concentrations of total cholesterol measured in DBS and serum samples. This concordance between studies is not just seen for total cholesterol with Quraishi et al. obtaining a correlation of r = 0.970 between triglyceride concentration measured in DBS and plasma samples (Quraishi et al. 2006) with subsequent studies achieving correlations of r = 0.838 and r = 944 (Lakshmy et al. 2012,2010). The ability to obtain a good measure of a given analyte with a strong correlation between plasma and DBS concentrations is not the only factor in ensuring that the two sample types are comparable; it is also important to ensure that the relationship between a given analyte and a clinical outcome measure present in the original blood sample are maintained in the DBS sample. For example if we consider measurement of activity of steraoyl-CoA desaturase using the ratio of PC(32:1) to PC(32:0), and either of PC(32:1) or PC(32:0) are diffuse differently across the sorbent or have differing extraction recoveries then this ratio will be changed giving false reading for the activity of this enzyme.

With this in mind the aims of this study were twofold. Firstly, we aimed to determine the comparability of results obtained from interrogating complex lipidomics data generated from DBS and plasma samples. We will do this by using random forest machine learning, which will be applied to lipid profile data generated from human plasma samples to predict the circulating concentration of four clinically important lipoproteins, triglyceride, HDL, LDL and total cholesterol. Subsequently we will measure these biomarkers in DBS samples and determine if these biomarkers can predict the concentration of these lipoproteins. We have chosen to use samples from the Dutch famine birth cohort and the Amsterdam Born Children and their Development cohorts rather than freshly collected samples as this better reflects the research cohorts that these developed approaches will be applied to. We have chosen to work with dried blood spot samples where the concentration of these lipoproteins was measured in a paired capillary blood sample rather than measured directly from the DBS. We have made this decision as we specifically want to DBS lipid profile data to predict the concentrations of these lipoproteins in blood and whilst measuring them in the DBS sample would provide an accurate surrogate of the blood concentration measuring it directly from the blood is preferable. Our second aim was to see if the estimated concentrations that we obtained from the DBS samples were accurate enough to be used in clinical practice and thus the bias of health care monitoring.

## Materials and methods

### Sample description

#### Dutch Famine Birth Cohort (DFBC)

The Dutch Famine Birth Cohort is a historical cohort of 2,414 men and women born in the Wilhemina Gasthuis hospital in Amsterdam between the 1st of November 1943 and 28th of February 1947. The selection procedures of this cohort have been described in detail previously (Lumey et al. 1993). In the present study, we analysed 777 plasma samples collected between 2002 and 2004 from individuals aged 55.7 to 60.8 years old, with all samples stored at -80 °C prior to analysis. The study was approved by the local Medical Ethics committee and conducted in accordance with the Helsinki declaration. All participants gave written informed consent.

#### Amsterdam Born Children and their Development (ABCD) cohort

The Amsterdam Born Children and their Development cohort is a large prospective birth cohort established in 2003–2004 in which mothers, fathers and their children are followed from pregnancy to adulthood, examining parental factors and their influence on the child’s health and development (Eijsden et al. 2011). Dried blood spots were collected in 2015–2016 when the children were between 11 and 13 years old with samples collected by numbing the participants finger before pricking it and dropping blood onto Whatman 903 cards filled with a solution of 5 mg butylated hydroxytoluene (BHT) per 10 ml ehthanol. Samples were subsequently dried at room temperature, catalogued and stored at − 80 °C prior to analysis. Lipid profiles were generated from 835 dried blood spot samples collected from children aged between 11.1 and 13.0 years (Eijsden et al. 2011). The study was approved by the local Medical Ethics committee and conducted in accordance with the Helsinki declaration. All participants and their parents gave written informed consent.

### Measurement of ‘clinical lipids’

In the Dutch Famine Birth Cohort, fasting blood was drawn for analysis of Low Density Lipoprotein (LDL)-cholesterol, High Density Lipoprotein (HDL)-cholesterol and triglyceride. HDL-cholesterol and triglyceride were measured using an enzymatic colorimetric reagent test using a P-800 Modular (Roche/Hitachi), with LDL-cholesterol calculated using the Friedewald formula and total cholesterol determined by combining the concentration of HDL and LDL cholesterol (Lumey et al. 1993). In the ABCD cohort samples were collected after a 3 h fasting period with capillary blood from a finger prick was collected within a capillary tube within 10 s with the sample subsequently transferred to the test cassette with TG, TC, HDL, and LDL concentration determined using the Cholestec LDX Analyzer (Eijsden et al. 2011).

### Preparation of plasma samples

Samples were extracted from plasma (which was stored at − 80 °C until assay) as described previously (Harshfield et al. 2019). In short: 100 µl of LC–MS grade water and 150 µl of internal standard mix (Table S1) were added to 15 µl of plasma in a 96 well glass coated plate prior to mixing for 10 s. Subsequently, 750 µl of LC–MS grade methyl-tertiary butyl ether (MTBE) and a further 200 µl of LC–MS grade water were added to each well before shaking for 10 s. Once mixed, plates were spun at 845×*g* for 2 min to achieve phase separation with 25 µl of the upper organic phase transferred to a new glass coated plate with 90 µl of MS-mix (7.5 mM ammonium acetate in IPA:CH_3_OH 2:1), which was subsequently added to each well.

### Preparation of dried blood spot samples

Dried blood spot samples were prepared as described previously (Koulman et al. 2014). In short: 100 µl of LC–MS grade water and 150 µl of internal standard mix (Table S1) were added to a 3.2 mm diameter dried blood spot in a 96 well glass coated plate prior to mixing for 10 s. Subsequently, 750 µl of LC–MS grade methyl-tertiary butyl ether (MTBE) and a further 200 µl of LC–MS grade water were added to each well before shaking for 10 s. Once mixed, plates were spun at 845 × *g* for 2 min to achieve phase separation with 150 µl of the upper organic phase transferred to a new glass coated plate and was dried down under a continuous stream of nitrogen. The dried samples were then reconstituted in 25 µl of MTBE to which 90 µl of MS-mix (7.5 mM ammonium acetate in IPA:CH3OH 2:1) was subsequently added to each well.

### Preparation of quality control samples

Three levels of quality controls samples 100% serum, 50:50 (*v:v*) serum:water and 25:75 (*v:v*) serum:water were prepared from a pooled serum sample with 15 µl extracted following the same procedure applied to DBS and plasma samples. In addition to being used to assess and correct plate to plate variance the quality control levels are also used to aid signal identification with real signals showing a linear increase in abundance with an increase in serum concentration.

### DIMS lipidomic profiling

Samples were infused into an Exactive Orbitrap (Thermo, Hemel Hempstead, UK) using a Triversa Nanomate (Advion, Ithaca, USA). Data collection began 20 s after the infusion began, initially analysing samples in the positive ionisation mode with an ionisation voltage of 1.2 kV applied. Data was acquired between 150 and 2000 m*/z* with a scan rate of 1 Hz giving a mass resolution of 65,000 at 400 m*/z*. A more detailed description of the instrument parameters can be found in Harshfield et al. (2019).

### Processing of lipidomic data

Raw data files were converted to.mzXML files using msConvert (ProteoWizard) (Chambers et al. 2012), and were subsequently processed in R (version 3.2.2) using an in house script to compare spectra against a list of 1649 lipid species, with a relative intensity and mass deviation value recorded for each lipid in every sample. We applied 4 filtering steps for quality control of the data and focus subsequent analysis on analytically robust signals. The first step was to remove lipids with a mean mass deviation between expected and recorded mass of greater than 5 ppm, the second step was to remove signals with an average intensity in the samples less than 5 times greater than in the blanks. The third step was to remove signals with 0 values in greater than 10% of samples. The final step was to remove lipids with an r < 0.9 in our QC dilution series.

In this study, the ‘discovery’ and ‘validation’ cohort were processed in parallel to ensure that datasets contained the same lipid variables. To control for potential differences in the volume of blood sampled from each blood spot all data was normalised to total signal intensity, this was done by calculating the total abundance of all of the lipid signals within a given sample then dividing the abundance of each of these signals by the total. After normalisation data was subsequently mean centred to allow for a direct comparison between the two cohorts. The data in the DBS cohort was filtered using the same steps as the plasma samples but was processed independently of the plasma samples as the composition of DBS samples will be different to that of plasma samples (Table 1).

**Table 1:** Clinical characteristics of the sample cohorts used in this study

	Dutch famine birth cohort	Amsterdam born children and their development cohort
Sample N^o^	777	835
Gender (% female)	54.2%	50.6%
Age (years)	58.3 ± 1.0	11.8 ± 0.4***
BMI	28.6 ± 4.9	17.6 ± 2.5***
TriG (mmol/L)	1.53 ± 1.00	0.97 ± 0.54***
HDL (mmol/L)	1.51 ± 0.42	1.48 ± 0.32***
LDL (mmol/L)	3.63 ± 1.00	2.17 ± 0.55***
Total Cholesterol (mmol/L)	5.83 ± 1.08	4.09 ± 0.65***

*BMI* body mass index, *HDL* high density lipoprotein, *LDL* low density lipoprotein, *TriG* triglyceride

*** < 0.0001

### Statistical analysis

Data analysis was performed for each of the four ‘clinical lipid’ markers (triglyceride, LDL, HDL and total cholesterol) independently. The analysis was performed in two stages, in the first stage of the analysis, we applied a random forest model to all of the data in the ‘discovery’ cohort (the DFBC) to identify a panel of lipid biomarkers capable of robustly predicting the concentration of the clinical biomarker. This was done by splitting the samples into training and test sets (samples split 70:30 respectively) and calculating a random forest model in the training set and assessing its performance in the test set. To determine the number of lipids to include in the predictive panel, iterative random forest models were calculated using the highest ranked variable from the ‘all data’ model first and then adding additional variables to each model (one at the time) until we achieved a model that performed as well as the model calculated using all lipids.

In the second stage, we determined if the panel of lipid biomarkers identified in the plasma samples from the DFBC could be used to predict the concentration of the clinical markers in the ‘validation’ cohort. This was done by dividing the DBS cohort (ABCD cohort) into a training and test set (70:30) and calculating a random forest model in the training set and assessing its predictive performance in the testing set based on the correlation between the measured and predicted concentration of the clinical biomarker.

The relationships between individual lipid species and given clinical biomarker concentrations were determined using generalised linear models (GLM) applied to the whole of the dataset. All models were calculated in ‘R’ (version 3.4.2). Controlling for type 1 errors was performed by determining if *p*-values passed a Bonferroni adjusted significance threshold of *p* = 0.0004 calculated based on all 125 lipids measured in this study.

## Results

In plasma samples 163 lipids from 11 classes passed quality control procedures, whilst 118 lipids from 11 classes were identified in the dried blood spot samples with 71% of the lipids measured in DBS also measured in plasma.

### Estimation of triglycerides concentration

**Fig. 1:**
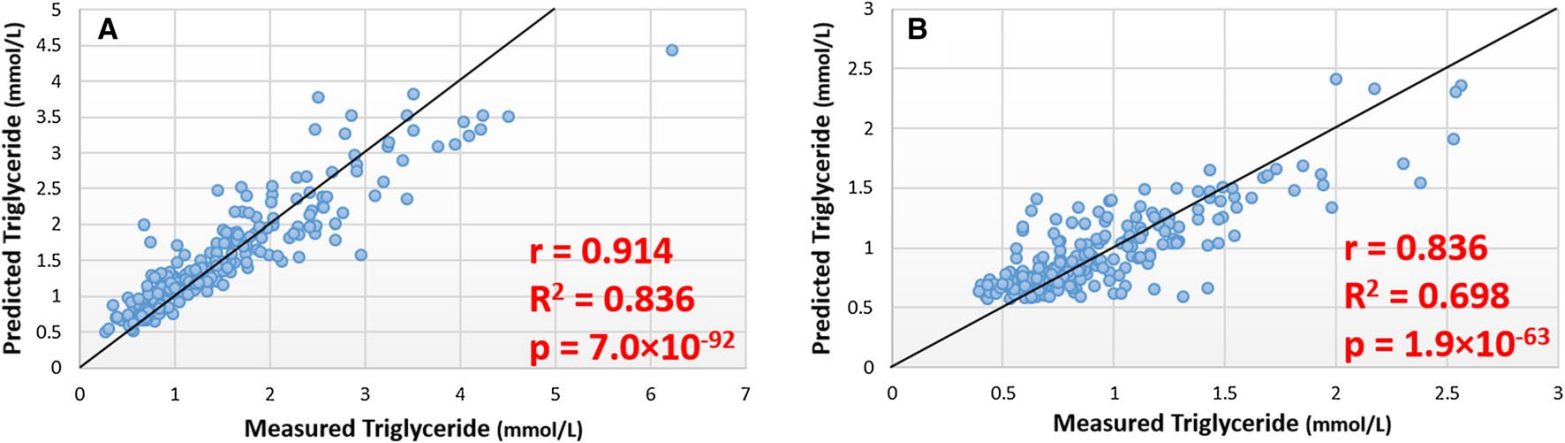
Scatter plots showing predicted vs. measured Triglyceride concentration in discovery and validation analysis. **a** Results of model trained and tested in ‘discovery’ cohort. **b** Results of models trained and tested in DBS ‘validation’ cohort using lipid markers identified in plasma

A panel of 12 lipids (Table S2) produced a model with a mean square of residual (MSR) of 0.199 in the training set (Table S2) and a correlation of r = 0.914 and p = 7.0 × 10^–92^ between estimated and measured triglyceride in the test set (Fig. 1a). With the exception of DG(34:2) and DG(36:2), which were not detected in lipid profiles generated from DBS samples the remaining 10 lipids were used to generate a random forest model with an MSR of 0.111 in the training set (Table S2), with estimated and measured triglyceride correlating with r = 0.836 and p = 1.9 × 10^–63^ in the test set (Fig. 1b). The samples in the test set of the DBS cohort were divided into quartiles based on their measured and their predicted TriG independently with the number of samples that were in the same quartile for both measured and predicted TriG concentration being recorded. The percentage overlap was higher in all quartiles than the 25% that we would expect to see by chance. It is also interesting to note that there was a higher percentage overlap in the 1st and 4th quartiles compared to the overlap in the 2nd and 3rd quartiles (Table 2).

**Table 2:** Comparison of relative distribution based on quartiles of measured clinical lipid concentration and the concentration predicted from dried blood spot samples

Dutch Famine Birth Cohort (plasma)					
		‘Desirable’	‘Borderline’	‘Poor’	
Triglyceride	Overlap*	144	16	34	
	% Overlap	90.5	64.0	72.3	
HDL	Overlap*	82	23	45	
	% Overlap	73.2	56.1	57.7	
LDL	Overlap*	30	147	13	
	% Overlap	85.7	89.1	41.9	
Total Cholesterol	Overlap*	3	135	12	
	% Overlap	100.0	64.6	63.1	

Amsterdam Born Children and their Development Cohort (dried blood spots)					
		1st Quartile	2nd Quartile	3rd Quartile	4th Quartile
Triglyceride	Overlap*	36	32	32	44
	% Overlap	60.0	53.3	53.3	73.3
HDL	Overlap*	37	24	21	35
	% Overlap	61.7	40.0	35.0	58.3
LDL	Overlap*	34	20	22	36
	% Overlap	56.7	33.3	36.7	60.0
Total Cholesterol	Overlap*	35	21	20	34
	% Overlap	58.3	35.0	33.3	56.7

Overlap* the number of samples with the measured and predicted lipid concentrations falling in the same quartile. **Triglyceride** ‘Desirable’ < 1.7 mmol/l, ‘Borderline’ 1.7–2.2 mmol/l, ‘Poor’ > 2.2 mmol/l, **HDL** ‘Desirable’ > 1.5 mmol/l, ‘Borderline’ 1.5–1.1 mmol/l, ‘Poor’ < 1.2.2 mmol/l, **LDL** ‘Desirable’ < 2.6 mmol/l, ‘Borderline’ 2.6–5.0 mmol/l, ‘Poor’ > 5.0 mmol/l, **Total cholesterol** ‘Desirable’ < 5.2 mmol/l, ‘Borderline’ 5.2–6.2 mmol/l, ‘Poor’ > 6.2 mmol/l

### Estimation of HDL concentration

**Fig. 2:**
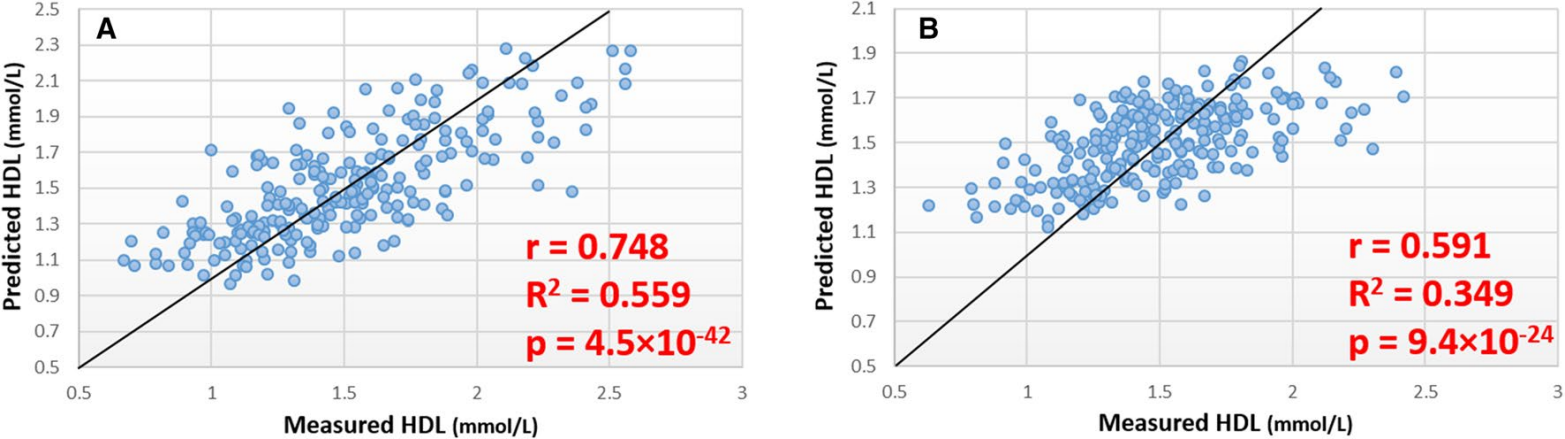
Scatter plots showing predicted vs. measured HDL concentration in discovery and validation analysis. **a** Results of model trained and tested in ‘discovery’ cohort. **b** Results of models trained and tested in DBS ‘validation’ cohort using lipid markers identified in plasma

In the discovery cohort a panel of 11 lipids (Table S3) was identified that generated a random forest model with an MSR of 0.082 in the training set (Table S2), with a correlation of r = 0.748 and p = 4.5 × 10^–42^ between estimated and measure HDL in the test set (Fig. 2a). All 11 of the lipids of panel identified in the discovery cohort of plasma samples were measured in the DBS samples with these lipid used to generate a random forest model with an MSR of 0.068 in the training set (Table S2), with a correlation of r = 0.591 and p = 9.4 × 10^–21^ between estimated and measure HDL concentration in the test set (Fig. 2b).

### Estimation of LDL concentration

**Fig. 3:**
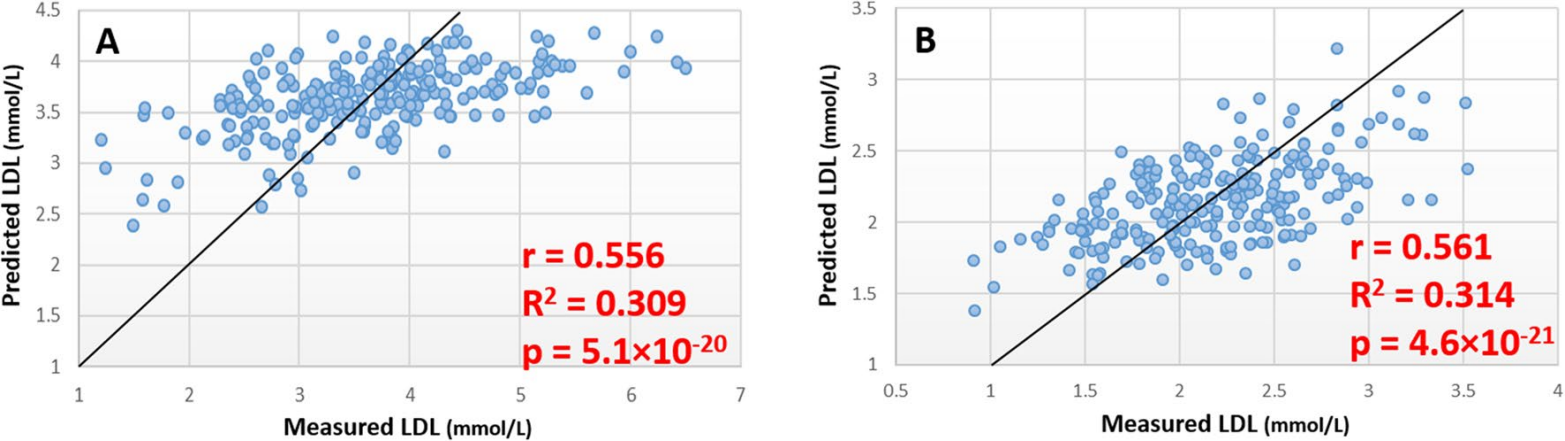
Scatter plots showing predicted vs. measured LDL concentration in discovery and validation analysis. **a** Results of model trained and tested in ‘discovery’ cohort. **b** Results of models trained and tested in DBS ‘validation’ cohort using lipid markers identified in plasma

In plasma samples random forest identified a 10 lipid panel of markers (Table S4) with an MSR of 0.811 in the training set (Table S2), with an estimated and measured LDL concentration correlating with r = 0.556 and p = 5.1 × 10^–21^ in the test set (Fig. 3a). In the DBS samples from the ABCD cohort CE(14:0) and TG(47:4) were not detected in the lipid profiles generated from DBS samples.A random forest model generated using the 8 remaining lipids from the predictive panel with an MSR of 0.231 in the training set (Table S2), and a correlation of r = 0.561 and p = 4.6 × 10^–21^ between estimated and measured LDL concentration in the test set (Fig. 3b).

The quartile distribution of LDL showed that the percentage overlap was higher in all quartiles than the 25% that we would expect to see by chance (Table 2).

### Estimation of total cholesterol concentration

**Fig. 4:**
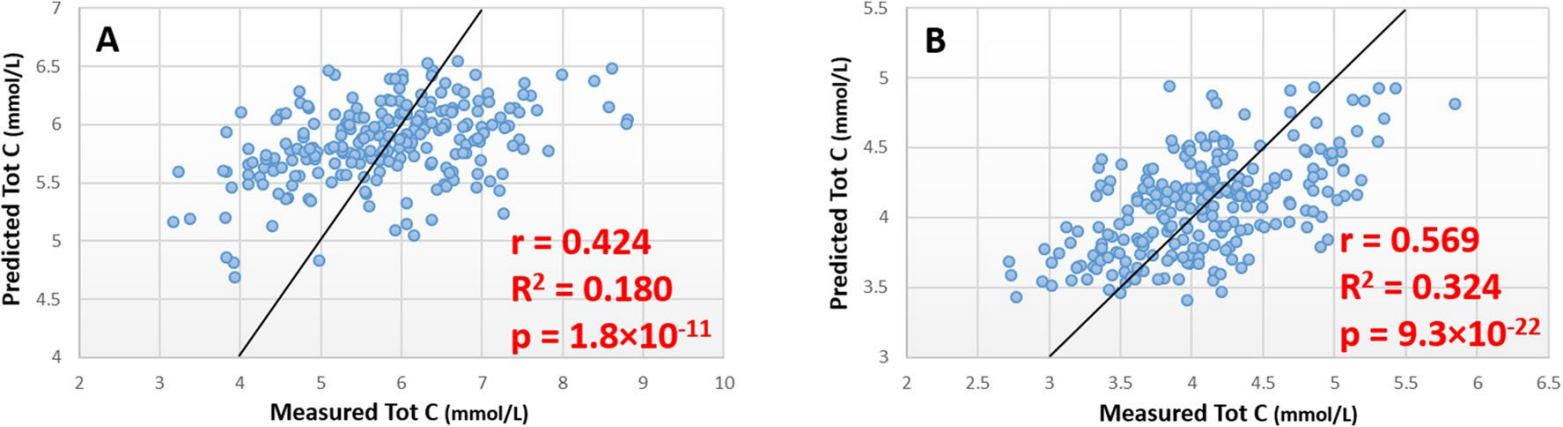
Scatter plots showing predicted vs. measured total cholesterol concentration in discovery and validation analysis. **a** Results of model trained and tested in ‘discovery’ cohort. **b** Results of models trained and tested in DBS ‘validation’ cohort using lipid markers identified in plasma

A panel of 11 lipids (Table S5) were used to generate a random forest model with an MSR of 1.05 in the training set (Table S2), and a correlation of r = 0.424 and p = 1.8 × 10^–11^ between estimated and measured total cholesterol in the test set (Fig. 4a). In the ABCD cohort, LPC(20:4), CE(14:0), CE(16:1) and PC-O(40:5) which form part of the predictive panel identified in plasma samples were not detected in the lipid profiles generated from DBS samples. A random forest model generated using the remaining 7 lipids with an MSR of 0.288 in the training set (Table S2), with estimated and measured total cholesterol concentration correlating with r = 0.569 and p = 9.3 × 10^–22^ (Fig. 4b). Again, as with the quartile distribution of the other lipids, the percentage overlap for total cholesterol was higher in all quartiles than the 25% that we would expect to see by chance (Table 2).

### Univariate analysis of lipid associations with lipoprotein concentration

Of the 44 lipid associations from the 4 predictive panels 8 (18%) were not measured in the DBS samples whilst 36 (82%) were successfully validated with a strong correlation (r = 0.917) observed between the correlation coefficients measured calculated between the individual lipid abundance and lipoprotein concentration in both plasma and DBS samples.

Of the 12 lipids used to predict triglyceride concentration all significantly associated with TriG concentration passing bonferroni correction at (p < 0.0004) in both DFBC and ABCD cohorts, except for DG(34:2) and DG(36:2) which were not measured in DBS samples (Table S2).

With the exception of PC(38:5) all significantly associated with HDL concentration passing bonferroni correction at (p < 0.0004) in both DFBC and ABCD cohorts (Table S3). Whilst not passing the bonferroni corrected p-threshold PC(38:5) was significantly (p < 0.05) associated with HDL concentration in both plasma and DBS samples (Table S3).

With the exception of CE(14:0) and TG(47:4) which were not measured in dried blood spot samples all lipids were significantly associated with LDL concentration in both sample types passing a bonferroni correct p-threshold (p < 0.0004) (Table S4).

Of the 11 lipids total cholesterol concentration all were significantly (p < 0.05) associated with total cholesterol concentration however only 7 passed a bonferroni correct p-threshold (p < 0.0004) with CE(18:2), PC-O(34:1), PC-O(40:5) and TG(56:5) falling short. In dried blood spots CE(14:0), CE(16:1), LPC(20:4) and PC-O(40:5) which were not measured, however the remaining 7 measured lipids were all significantly associated with total concentration and passed multiple comparison correction based on bonferroni correct p-threshold (p < 0.0004) with all of these lipids also exhibiting a stronger association than is observed between these lipids and total cholesterol concentration than is seen in the discovery cohort (Table S5).

### Stratification based on estimated lipoprotein concentration

Plasma samples were stratified into the clinical risk categories based on their estimated lipoprotein concentration (Table 2). Total classification accuracies of 83.9%, 64.9%, 82.2%, and 64.9% were obtained for triglyceride, HDL, LDL and total cholesterol respectively (Table 2). Classification accuracy was not equal across the groups for any of the lipoproteins, with the risk category with the most samples in it having the highest accuracy with the exception of the ‘low risk’ total group which only contained 3 samples.

Stratification of DBS samples in the ABCD cohort was based on quartile rather than clinical cut-offs as the samples came from adolescents only 3% of the measurements fell outside of the healthy range. Classification accuracy was not equal across the quartiles with all of the lipoproteins showing better classification accuracy in the 1st and 4th quartiles (Table 2).

## Discussion

To our knowledge this is the first study to compare results obtained from DBSs and plasma samples in complex lipidomics studies and to determine that if we tried to answer the same biological question in both sample types would we achieve the same answer. Whilst we measured more lipids from the plasma samples (163 compared to 118) a high proportion (71%) of the lipids measured in the DBS samples were also measured in plasma, with this lower number of lipids likely the result of less biological material being obtained from a single DBS punch than was extracted for the plasma samples.

Applying random forest machine learning to the lipid profile data generated from the plasma samples we were able to obtain good estimates (r > 0.7) of the concentration of triglyceride (12 lipid panel) and HDL (11 lipid panel) and modest estimates (r > 0.4) of LDL (10 lipid panel) and total cholesterol (11 lipid panel) When these lipids were measured in an independent cohort of DBS samples 2 of the TG markers were not measured whilst the other 10 validated the association seen in the plasma samples. For HDL all 11 lipids validated, for LDL 2 were not measured and 8 validated and for total cholesterol 4 lipids were not measured in DBS while the remaining 7 lipids validated between cohorts. Using these panels of lipid biomarkers identified in plasma samples we were also able to use them to obtain a good estimate of triglyceride concentration and modest estimates of HDL, LDL and total cholesterol concentrations. This begins to suggest that dried blood spots can be used as an alternative to plasma samples and that the process of adding and removing the sample from the card is not altering the composition of the lipidome and its association with relationship with outcome measures, in this case the concentration of the four ‘clinical lipids’. This finding is keeping with previous reports that have shown DBS as a viable alternative (Koulman et al. 2014; Mahajan et al. 2018) however this is the first study to directly this comparability.

We also wanted to see if the concentration estimates generated from both plasma and DBS samples were accurate enough to be used in clinical practice. Knowing a patients exact concentration of TriG, HDL, LDL or total cholesterol is less important than knowing their relative concentration (https://www.nhs.uk/conditions/high-cholesterol/cholesterol-levels/; Nantsupawat et al. 2019), i.e. if they fall outside of the healthy range, with a well-defined set of cut-offs described for each of the four ‘clinical lipids’ (https://www.nhs.uk/conditions/high-cholesterol/cholesterol-levels/; Nantsupawat et al. 2019). In plasma our estimated concentrations classified people into the correct clinical category with accuracies of 83.9%, 64.9%, 82.2%, and 64.9% for triglyceride, HDL, LDL and total cholesterol respectively. However, it is generally accepted that clinical biomarkers and test should have a sensitivity and specificity over 90% (“Biomarkers: portents of malignancy”). So whilst these accuracies are all significantly higher than we would expect to see if we assigned individuals to groups randomly, but they are not good enough to be used in clinical practice. In the DBS cohort, looking at clinical cut-offs was inappropriate as the samples came from children with only 3% of the measurements falling outside of the healthy range, instead we looked at the relative distribution based on quartiles. As with plasma samples this is higher classification accuracy than we would expect if we assigned individuals to groups randomly but it still falls well short of the 90% sensitivity and specificity required for clinical practice.

Whilst the estimates that we have obtained in this study are not currently accurate enough for clinical use there does appear to be room for improvement. Looking at both plasma and DBS sample it can be seen that the risk category that most samples belong to being the most accurately classified as there was an overall bias with an significant over estimation of concentrations in this range (Table 2). This bias occurs because during the training phase the model identifies the optimum parameters to obtain the best prediction accuracy possible. So if the samples are clustered at the bottom of the concentration range, then the model will be biased towards predicting low concentrations as this will increase prediction accuracy for example in a situation where we have no information about a sample based on probability it is more likely to have a low concentration than a high one. Triglyceride has a more even distribution across the measure concentration range in both plasma and DBS (and as a result of this we for this lipoprotein we see a stronger correlation between estimated and measured concentration as well as a higher accuracy when assigning individuals to clinical groups relative to the other three lipoproteins in both plasma and DBS samples.

Whilst fewer lipids were measured in dried blood spots relative to plasma samples lipid markers used to predict the concentration of lipoproteins in plasma also predict their concentration when measured in DBS samples. This demonstrates that dried blood spots have the same association between the lipid profile of the sample and the clinical outcome.

## Electronic supplementary material

Below is the link to the electronic supplementary material.

Supplementary file1 (DOCX 29 kb)

## Data Availability

Data is available by request from the cohort owner.
