# Supplementary material for: Combining lipidomics and machine learning to measure clinical lipids in dried blood spots
Source: Metabolomics. 2020 Jul 24;16(8):83. doi: 10.1007/s11306-020-01703-0 (PMC7381462; doi:10.1007/s11306-020-01703-0)
Supplement: Supplementary file 1 — Supplementary file1 (DOCX 29 kb) [file 11306_2020_1703_MOESM1_ESM.docx]

**Supplemental Table 1 list of internal standards added during sample preparation.**

| **Lipid Class** | **Lipid Chemistry** | **Concentration (µM)** | **Supplier** |
| --- | --- | --- | --- |
| Cholesteryl ester | CE(18:0-d_6_ ) | 3.80 | QMX |
| Ceramide | C16-d_31_ Ceramide | 4.40 | AVANTI |
| Fatty acid | C15:0-d_29_ FA | 9.23 | QMX |
| Fatty acid | C17:0-d_33_ FA | 8.25 | QMX |
| Fatty acid | C20:0-d_39_ FA | 7.13 | QMX |
| *lyso*-Phosphatidylcholine | *lyso*PC(C14:0)-d_42_ | *5.93* | QMX |
| Phosphatidic acid | PA(C16:0-d_31_/C18:1) Na^+^ salt | 3.55 | AVANTI |
| Phosphatidylcholine | PC(C16:0-d_31_/C18:1) | 3.18 | AVANTI |
| Phosphatidylethanolamine | PE(C16:0-d_31_/C18:1) | 3.35 | AVANTI |
| Phosphatidylglycerol | PG(C16:0-d_31_/C18:1) Na^+^ salt | 3.20 | AVANTI |
| Phosphatidylinositol | PI(C16:0-d_31_/C18:1) NH_4_^+^ salt | 1.13 | AVANTI |
| Phosphatidylserine | PS(C16:0-d_62_) Na^+^ salt | 3.40 | AVANTI |
| Sphingomyelin | SM(C16:0-d_31_) | 2.04 | AVANTI |
| Triglyceride | TG(45:0-d_29_) | 2.93 | QMX |
| Triglyceride | TG(48:0-d_31_) | 2.78 | QMX |
| Triglyceride | TG(54:0-d_35_) | 2.50 | QMX |

**Supplemental Table 2 Measures of training performance reported as mean square of residuals and correlation between estimated and actual values for models used to estimate the concentration of 4 lipoproteins in both plasma and DBS samples.**

| **Dutch Famine Birth Cohort (Plasma)** | | |
| --- | --- | --- |
|  | **MSR** | **R^2^** |
| Triglyceride | 0.199 | 0.972 |
| HDL | 0.082 | 0.949 |
| LDL | 0.811 | 0.967 |
| Total Cholesterol | 1.051 | 0.977 |
| **Amsterdam Born Children and their Development Cohort (DBS)** | | |
|  | **MSR** | **R^2^** |
| Triglyceride | 0.111 | 0.954 |
| HDL | 0.068 | 0.959 |
| LDL | 0.231 | 0.964 |
| Total Cholesterol | 0.288 | 0.958 |

HDL; high density lipoprotein, LDL; low density lipoprotein, MSR; mean square of residuals.

**Supplemental Table 3 univariate associations of measured plasma triglyceride concentration and the lipids used to make the prediction of triglyceride level.**

|  | **Dutch Famine Birth Cohort** | | **ABCD Cohort** | |
| --- | --- | --- | --- | --- |
|  | p-value | Correlation | p-value | Correlation |
| DG(34:2) | 2.0×10^-16*^ | 0.646 | NM | NM |
| TG(50:3) | 2.0×10^-16*^ | 0.701 | 2.0×10^-16*^ | 0.711 |
| TG(50:2) | 2.0×10^-16*^ | 0.732 | 2.0×10^-16*^ | 0.746 |
| DG(36:2) | 2.0×10^-16*^ | 0.499 | NM | NM |
| DG-H_2_O(34:1) | 2.0×10^-16*^ | 0.700 | 2.0×10^-16*^ | 0.747 |
| SM(42:2) | 2.0×10^-16*^ | -0.672 | 2.0×10^-16*^ | -0.444 |
| DG(36:3) | 2.0×10^-16*^ | 0.398 | 2.0×10^-16*^ | 0.481 |
| SM(34:1) | 2.0×10^-16*^ | -0.652 | 2.0×10^-16*^ | -0.485 |
| TG(52:2) | 2.0×10^-16*^ | 0.670 | 2.0×10^-16*^ | 0.694 |
| TG(52:3) | 2.0×10^-16*^ | 0.522 | 2.0×10^-16*^ | 0.647 |
| CE(18:1) | 2.0×10^-16*^ | -0.641 | 2.0×10^-16*^ | -0.183 |
| PC-O(34:1) | 2.0×10^-16*^ | -0.655 | 2.0×10^-16*^ | -0.421 |

p-values and correlation coefficients showing the association between individual lipids and plasma triglyceride concentrations in each of the three cohorts analysed. *2.0×10^-16^ is the lowest p-value returned by the R package used to calculate these generalised linear models. CE; cholesterylester, DG; diglyceride, PC-O; phosphatidylcholine (ether linked), SM; sphingomyelin, TG; triglyceride.

**Supplemental Table 4 univariate associations of measured plasma HDL concentration and the lipids used to make the prediction of HDL level.**

|  | **Dutch Famine Birth Cohort** | | **ABCD Cohort** | |
| --- | --- | --- | --- | --- |
|  | p-value | Correlation | p-value | Correlation |
| TG(52:3) | 2.0×10^-16*^ | -0.555 | 2.0×10^-16*^ | -0.376 |
| DG-H20(34:1) | 2.0×10^-16*^ | -0.588 | 2.0×10^-16*^ | -0.298 |
| PC(34:3) | 2.0×10^-16*^ | 0.470 | 9.0×10^-5^ | 0.116 |
| DG-H20(34:2) | 2.0×10^-16*^ | -0.602 | 2.0×10^-16*^ | -0.354 |
| DG-H20(36:2) | 2.0×10^-16*^ | -0.528 | 2.0×10^-16*^ | -0.301 |
| PC(38:5) | 2.0×10^-16*^ | 0.500 | 0.019 | 0.082 |
| TG(52:2) | 2.0×10^-16*^ | -0.532 | 2.2×10^-15^ | -0.275 |
| TG(54:2) | 2.0×10^-16*^ | -0.459 | 6.4×10^-5^ | -0.141 |
| TG(52:4) | 2.0×10^-16*^ | -0.504 | 3.4×10^-11^ | -0.328 |
| PC-O(34:3) | 2.0×10^-16*^ | 0.522 | 2.0×10^-16*^ | 0.397 |
| TG(53:3) | 2.0×10^-16*^ | -0.470 | 9.0×10^-14^ | -0.259 |

p-values and correlation coefficients showing the association between individual lipids and plasma HDL concentrations in each of the three cohorts analysed. *2.0×10^-16^ is the lowest p-value returned by the R package used to calculate these generalised linear models. DG; diglyceride, PC; phosphatidylcholine, PC-O; phosphatidylcholine (ether linked), TG; triglyceride.

**Supplemental Table 5 univariate associations of measured plasma LDL concentration and the lipids used to make the prediction of LDL level.**

|  | **Dutch Famine Birth Cohort** | | **ABCD Cohort** | |
| --- | --- | --- | --- | --- |
|  | p-value | Correlation | p-value | Correlation |
| CE(18:2) | 9.6×10^-12^ | 0.242 | 2.0×10^-16*^ | 0.313 |
| CE(18:3) | 1.1×10^-5^ | 0.157 | 2.0×10^-16*^ | 0.322 |
| CE(14:0) | 2.2×10^-9^ | 0.214 | NM | NM |
| CE(20:3) | 5.9×10^-15^ | 0.277 | 2.0×10^-16*^ | 0.209 |
| TG(47:4) | 1.8×10^-11^ | 0.239 | NM | NM |
| CE(18:1) | 3.1×10^-7^ | 0.183 | 3.0×10^-9^ | 0.271 |
| PC-O(34:1) | 4.2×10^-6^ | 0.190 | 2.9×10-^12^ | -0.144 |
| CE(20:5) | 3.1×10^-5^ | 0.150 | 2.1×10^-9^ | 0.331 |
| SM(42:1) | 1.5×10^-10^ | 0.228 | 7.6×10^-9^ | 0.267 |
| TG(48:4) | 1.7×10^-10^ | 0.228 | 3.0×10^-9^ | 0.271 |

p-values and correlation coefficients showing the association between individual lipids and plasma LDL concentrations in each of the three cohorts analysed. *2.0×10^-16^ is the lowest p-value returned by the R package used to calculate these generalised linear models. CE; cholesterylester, PC-O; phosphatidylcholine (ether linked), SM; sphingomyelin, TG; triglyceride.

**Supplemental Table 6 univariate associations of measured plasma total cholesterol concentration and the lipids used to make the prediction of total cholesterol level.**

|  | **Dutch Famine Birth Cohort** | | **ABCD Cohort** | |
| --- | --- | --- | --- | --- |
|  | p-value | Correlation | p-value | Correlation |
| CE(18:3) | 1.4×10^-4^ | 0.134 | 7.5×10^-6^ | 0.157 |
| LPC(20:4) | 9.7×10^-5^ | -0.139 | NM | NM |
| CE(14:0) | 1.4×10^-6^ | 0.173 | NM | NM |
| CE(20:3) | 6.4×10^-5^ | 0.144 | 2.0×10^-16*^ | 0.314 |
| CE(16:1) | 6.0×10^-4^ | 0.123 | NM | NM |
| PC-O(40:5) | 0.008 | -0.096 | NM | NM |
| CE(20:5) | 6.0×10^-4^ | 0.124 | 1.4×10^-11^ | 0.235 |
| LPC(18:2) | 4.2×10^-5^ | -0.147 | 9.2×10^-8^ | -0.187 |
| CE(18:2) | 0.007 | 0.097 | 2.0×10^-13^ | 0.255 |
| TG(56:5) | 0.002 | -0.112 | 4.5×10^-5^ | 0.143 |
| PC-O(34:1) | 0.007 | -0.097 | 2.0×10^-16*^ | -0.325 |

p-values and correlation coefficients showing the association between individual lipids and plasma total cholesterol concentrations in each of the three cohorts analysed. *2.0×10^-16^ is the lowest p-value returned by the R package used to calculate these generalised linear models. CE; cholesterylester, LPC; lysophosphatidylcholine, PC-O; phosphatidylcholine (ether linked), TG; triglyceride.
